# Collagen molecular organization preservation in human fascia lata and periosteum after tissue engineering

**DOI:** 10.3389/fbioe.2024.1275709

**Published:** 2024-04-03

**Authors:** Julia Vettese, Julie Manon, Antoine Chretien, Robin Evrard, Lies Fievé, Thomas Schubert, Benoît G. Lengelé, Catherine Behets, Olivier Cornu

**Affiliations:** ^1^ Neuromusculoskeletal Lab (NMSK), Institut de Recherche Expérimentale et Clinique (IREC), UCLouvain, Brussels, Belgium; ^2^ Morphology Lab (MORF), IREC, UCLouvain, Brussels, Belgium; ^3^ Centre de Thérapie Cellulaire et Tissulaire Locomoteur, Cliniques Universitaires Saint-Luc, Brussels, Belgium; ^4^ Department of Orthopaedic and Trauma Surgery, Cliniques Universitaires Saint-Luc, Brussels, Belgium; ^5^ Department of Plastic and Reconstructive Surgery, Cliniques Universitaires Saint-Luc, UCLouvain, Brussels, Belgium

**Keywords:** extracellular matrix, collagen, crosslinks, decellularization, sterilization

## Abstract

Large bone defect regeneration remains a major challenge for orthopedic surgeons. Tissue engineering approaches are therefore emerging in order to overcome this limitation. However, these processes can alter some of essential native tissue properties such as intermolecular crosslinks of collagen triple helices, which are known for their essential role in tissue structure and function. We assessed the persistence of extracellular matrix (ECM) properties in human fascia lata (HFL) and periosteum (HP) after tissue engineering processes such as decellularization and sterilization. Harvested from cadaveric donors (N = 3), samples from each HFL and HP were decellularized following five different chemical protocols with and without detergents (D1-D4 and D5, respectively). D1 to D4 consisted of different combinations of Triton, Sodium dodecyl sulfate and Deoxyribonuclease, while D5 is routinely used in the institutional tissue bank. Decellularized HFL tissues were further gamma-irradiated (minimum 25 kGy) in order to study the impact of sterilization on the ECM. Polarized light microscopy (PLM) was used to estimate the thickness and density of collagen fibers. Tissue hydration and content of hydroxyproline, enzymatic crosslinks, and non-enzymatic crosslinks (pentosidine) were semi-quantified with Raman spectroscopy. ELISA was also used to analyze the maintenance of the decorin (DCN), an important small leucine rich proteoglycan for fibrillogenesis. Among the decellularization protocols, detergent-free treatments tended to further disorganize HFL samples, as more thin fibers (+53.7%) and less thick ones (−32.6%) were recorded, as well as less collagen enzymatic crosslinks (−25.2%, *p* = 0.19) and a significant decrease of DCN (*p* = 0.036). GAG content was significantly reduced in both tissue types after all decellularization protocols. On the other hand, HP samples were more sensitive to the D1 detergent-based treatments, with more disrupted collagen organization and greater, though not significant loss of enzymatic crosslinks (−37.4%, *p* = 0.137). Irradiation of D5 HFL samples, led to a further and significant loss in the content of enzymatic crosslinks (−29.4%, *p* = 0.037) than what was observed with the decellularization process. Overall, the results suggest that the decellularization processes did not significantly alter the matrix. However, the addition of a gamma-irradiation is deleterious to the collagen structural integrity of the tissue.

## 1 Introduction

Bone regeneration is a well-regulated process requiring the recruitment of skeletal stem/progenitor cells (SSPCs) from adjacent tissues. Among them periosteum is a connective tissue that surrounds the external surface of bones and has been widely described as physiologically essential for bone development and healing (Duchamp de Lageneste and Colnot, 2019; Julien et al., 2022; Perrin and Colnot, 2022). This membrane consists of a fibrous outer layer composed of type I collagen and a highly cellular inner layer composed of fibroblasts, osteoprogenitor cells and mesenchymal stem cells (Squier, Ghoneim, and Kremenak, 1990; Dwek, 2010). However, in case of critical bone defect after a massive trauma, a tumoral resection or an osteomyelitis, the surrounding soft tissues, such as periosteum, are usually damaged and the normal physiological healing capacity is insufficient (Vidal et al., 2020).

Current strategies for the reconstruction of large bone defects exploit surgical approaches such as Ilizarov’s (Aktuglu, Erol, and Vahabi, 2019) or Masquelet’s induced-membrane (Masquelet and Begue, 2010) techniques. However, these methods require lengthy and tedious processing. Bone repair therefore remains a major concern for orthopedic surgeons (Evans, Chang, and Knothe Tate, 2013; Delloye et al., 2014; Dalisson et al., 2021) which has led to the emergence of a series of alternative tissue engineering approaches in recent decades (Chen et al., 2015; Lou et al., 2021; Manon et al., 2023). Among these, Manon et al. recently showed the suitability of using human fascia lata matrices as a periosteal-like scaffold for bone regeneration, mainly through their collagen type I composition and appropriate mechanical properties (Manon et al., 2022; Manon et al., 2023). The human fascia lata (HFL) is a dense connective tissue made up of three layers of collagen fiber bundles with little cellularity (Pancheri et al., 2014; Blottner et al., 2019).

Along with bone marrow and muscle, the periosteum is an important source of skeletal stem/progenitor cells (SSPCs) with a high potential for bone regeneration (Zhang et al., 2005; Duchamp de Lageneste et al., 2018). These progenitors reside in a rich environment formed by the periosteal extracellular matrix, where cellular and molecular crosstalk is crucial to achieve bone regeneration (Julien et al., 2022). This three-dimensional scaffold therefore not only provides a structural and mechanical support (Costa-Almeida et al., 2015; Hussey, Dziki, and Badylak, 2018; Sainio and Järveläinen, 2020) but is also a modulator of tissue homeostasis (Theocharis et al., 2016). Consequently, any alteration of this structure can lead to the disruption of tissue function (Theocharis et al., 2016). Collagen constitutes the major ECM component (Shoulders and Raines, 2009; Yamauchi and Sricholpech, 2012) and plays a key role in maintaining biological and architectural integrity of tissues as well as providing physical support (Dong and Lv, 2016). Lin et al. (2018) further emphasized this point by revealing that cell-free collagen scaffold of decellularized periosteum alone efficiently promotes orthotopic remodeling of skeletal defects *in vivo* (Lin et al., 2018).

Fibril forming collagen type I, the most prevalent one, provides tensile strength to tissues (Theocharis et al., 2016) through its complex hierarchical structure. Encoded by the *COL1A1* and *COL1A2* genes, collagen precursors undergo series of post-translational modifications (PTMs) to form mature molecules (Gjaltema and Bank, 2017; Rappu et al., 2019), which are stabilized by two main types of intermolecular crosslinks important for their assembly and for collagen functions (Yamauchi and Sricholpech, 2012). First, enzymatic crosslinks, considered a post-translational modification of lysine residues, are mediated by the lysyl hydroxylases and lysyl oxidases through a highly controlled mechanism. Initially formed as immature divalent crosslinking (e.g., dehydrodihydroxylysinonorleucine), some are converted into mature trivalent crosslinks (e.g., pyridinoline) (Thorpe, 2010; Yamauchi and Sricholpech, 2012; Saito and Marumo, 2015; Kamml et al., 2023). Secondly, non-enzymatic crosslinks result from a glycation process forming advanced glycation end products (AGEs), among which pentosidine is the most frequently characterized (Paul and Bailey, 1996; Pritchard and Willett, 2017). All AGEs are formed by a Lysine-Arginine or Lysine-Lysine interaction and predominantly accumulate in collagen because of its low turnover. Their emergence leads to an increase in connective tissue rigidity and insolubility (Paul and Bailey, 1996; Gautieri et al., 2014). Finally, interfibrillar bridges also result from the interactions of non-collagenous components between glycosaminoglycans (GAG)-decorin (DCN) complexes of adjacent fibrils (Scott, 2003; Thorpe, 2010; Costa-Almeida et al., 2015). DCN is a member of class 1 small leucine-rich proteoglycans (SLRPs) essential for the regulation of various tissular processes (Halper, 2014; Gubbiotti et al., 2016) but most notably studied for its involvement in collagen fibrillogenesis and matrix stability. This protein also acts as a reservoir of growth factors and cytokines (Gubbiotti et al., 2016; Zhang et al., 2018).

In the present study harvested human periosteum (HP) and HFL samples were treated with several protocols of decellularization (Manon et al., 2023) as well as by irradiation. Whereas the general microstructure, composition, and biosafety of the ECM-derived scaffold had received considerable attention, a special analysis of collagen *per se* is required as the main component contributing to matrix-cell crosstalks and, hence, to the global integrity of the periosteum. Therefore, since decellularization and sterilization processes can have a negative impact on the ECM, the aim of the present study was to characterize the collagen scaffold obtained after these engineering processes. Here, we hypothesized and sought to confirm the global persistence of important ECM components of HP and HFL samples after chemical decellularization and sterilization, and therefore a persistence of their functional and structural properties. The ultimate goal of this study is to confirm the maintenance of HFL matrices properties in order to consider their future use as periosteal scaffold substitutes to promote bone regeneration.

## 2 Materials and methods

### 2.1 Sample collection and processing

HFL and HP were harvested from deceased donors (3 donors of average 86.81 ± 3.7 years) of the Human Anatomy Department of UCLouvain (Body donation service, IRB00008535, Brussels, Belgium) following the same procedure as previously described (Manon et al., 2022) and were subjected to five chemical decellularization (D) protocols (Table 1) performed under continuous agitation (N = 3 donors x 2 tissues x 5 protocols = 30 analyzes). The soap-based detergent protocols, D1 to D4, consisted of different combinations of Triton, Sodium dodecyl sulfate (SDS) and Deoxyribonuclease (DNase). Triton is a non-ionic detergent that disturbs the lipid-lipid and protein-DNA interactions. SDS is an ionic detergent that denatures protein structure and solubilizes nuclear and cellular membranes. DNase treatment is employed to cleave internucleotide bonds and eliminate undesirable cellular waste. In protocol D1, tissues were maintained for 12 h in SDS, followed by 4 h in Triton. The sequence was reversed for D2, with 12 h in Triton and by 4 h in SDS. For protocols D3 and D4, the sequence was the same as for D1 and D2, respectively, with an increase in the duration of the first treatment step maintained for 24 h instead of 12 h (SDS and Triton, respectively). Each protocol ended with a 12 h DNase treatment. D5 protocol is the one routinely used in the institutional tissue bank (van Steenberghe et al., 2017; Manon et al., 2023). In brief, it includes sequential immersion in pure acetone to degrease, ether to neutralize the previous compounds and 70° ethanol to further degrease. Following a washing step, sample are immersed in a hypertonic solution of NaCl (7%) and NaOH to finally been placed in H_2_O_2_ (7.5%). Quality controls were performed to confirm the full decellularization and to measure the pH and traces of H_2_O_2_ and acetone. To assess the efficacy of decellularization protocols, microscopic analyses were performed on DAPI and Hematoxylin and Eosin (HE) stained slices first. Then, 3 random samples of 25 mg were harvested from native and decellularized tissues of all protocols, and freeze-dried. The DNA content was measured using the Quant-iT PicoGreen DNA assay kit (ThermoFisher, Waltham, MA, United States) just after being extracted using the DNeasy^®^ Blood & Tissue kit (Qiagen, Hilden, Germany). The very precise steps are previously described (Manon et al., 2022; Manon et al., 2023). Samples were then stored at −80°C.

**TABLE 1:** Decellularization protocols (D), from D1 to D5.

	Decellularization protocols
D1	12 h SDS 4 h Triton 12 h DNase
D2	12 h Triton 4 h SDS 12 h DNase
D3	24 h SDS 4 h Triton 12 h DNase
D4	24 h Triton 4 h SDS 12 h DNase
D5	Acetone, Ether, ethanol, NaCl (7%), NaOH, H_2_O_2_

SDS, sodium dodecyl sulfate; DNase, deoxyribonuclease.

Samples of decellularized HFL (D1-D5) were then frozen and sterilized by gamma(γ)-irradiation (HFLi, HFL irradiated) under dry ice using a minimal exposure dose of 25 kGy (IBA Mediris, Fleurus, Belgium), the recommended standard sterilization method for clinical applications (Dziedzic-Goclawska et al., 2005; Seto, Gatt, and Dunn, 2008; Harrell et al., 2018).

To perform the various analyses, samples were randomly taken from the entire decellularized scaffold to consider the whole tissue heterogeneity.

### 2.2 Polarized light microscopy

After paraffin histology processing, sirius red (SR) staining, an acidic hydrophilic stain, was used to highlight and evaluate qualitative modification in collagen organization (n_(HFL)_ = 4 samples/protocol, n_(HP)_ = 3 samples/protocol, with 3 sections per native samples and 1 per decellularization protocol). Deparaffinized and rehydrated sections were immersed in 1% Phosphomolybdic acid and then in 0.1% Sirius Red solution for 2 h. Sections were then incubated in hydrochloric acid before washing and mounting steps, then imaged under polarized light. Parallelly bound to collagen fibrils, this dye intensifies the birefringence of the samples which is manifested by shades of color under polarized light, each associated with properties of fiber organization. Thin fibers (diameter ≤0.8 µm) with a more scattered histoarchitecture are characterized by a weak birefringence and are detected in green/yellow while thick (diameter between 1.6–2.4 µm) organized ones appeared as shades of red and orange (Dayan et al., 1989). These parameters are quantified in order to highlight a possible alteration in collagen organization.

SR-stained sections were first imaged using the Axioplan microscope (ZEISS) with a 10-fold magnification. Linear polarization of light was obtained by using two filters that allow light to pass only in one direction of vibration: the polarizer and the analyzer, placed perpendicularly (Rittié, 2017). All slides were examined at the same time to ensure consistency of the experiment and were digitalized using the Nikon Digital sight DS SMC camera with NIS-Element BR 3.0 software. The average of five different acquisitions was assessed for each HFL section to cover the whole tissue surface, and three for HP, the surface of the sections being smaller. Collagen organization was then estimated with ImageJ software (Schneider, Rasband, and Eliceiri 2012) as a surface percentage of each color with a constructed macro (IREC imaging platform, 2IP). The analysis displayed green, yellow, orange, and red polarization colors.

To further validate our findings, whole slide images were then acquired with the ZEISS Axio Scan.Z1 Slide scanner under polarized light in order to approximate the proportion of each fiber type. These acquisitions were analyzed with QuPath (Bankhead et al., 2017) calibrated to detect the different shades of colors. With this approach, unlike with the previous one, only green, orange, and red colors are detected.

### 2.3 Raman spectroscopy

#### 2.3.1 Spectra acquisition

Molecular composition of fresh frozen and processed HFL, HFLi and HP samples (*n* = 3/protocol for each type of samples) was investigated using Raman spectroscopy (Renishaw confocal Raman inVia Quontor). Spectra of each sample were obtained as the average of 3 consecutive spectra randomly distributed over the sample surface. Each spectrum was collected using a 785 nm monochromatic laser beam with a power of 50 mW, a 50-fold magnification and the Renishaw Centrus 21KP95 detector. Two types of spectra ranges were recorded.

Acquisitions were first performed with a static mode in a wavelength range of 700–1800 cm^−1^ with an integration time of 10 s and 15 accumulations. The presence of non-enzymatic crosslinks (pentosidine, 1345 cm^−1^) and hydroxyproline (872 cm^−1^) was normalized to the amount of tissue using the proline peak (920 cm^−1^). Enzymatic crosslink level is assessed by the ratio of mature to immature crosslinks (pyridinoline/dehydrodihydroxylysinonorleucine, 1670/1690 cm^−1^). A 1657 cm^−1^ supplementary band was added for the Amide-I peak-fit model of HP. This lipid vibration band was used to correct its potential interference on the measure of enzymatic crosslinks (Mandair et al., 2020).

To estimate the collagen-bound water content, a second wavelength range of 2700–3800 cm^−1^ was recorded with extended mode, 10 s integration time, and 6 accumulations. Three parameters were considered: organic matrix-related water (3243 cm^−1^), amine (NH) groups beside organic matrix-related water (3333 cm^−1^) and hydroxide (OH) groups of hydroxyproline beside collagen-related water (3457 cm^−1^). Each of these parameters was normalized to the amount of organic matrix (mainly collagen) assessed at a wavelength of 2940 cm^−1^ (Unal and Akkus, 2015).

#### 2.3.2 Spectra processing

Raman spectra were processed using two different softwares: the Windows®-based Raman Environment (WiRE 3.5, Renishaw) and MATLAB PLS toolbox (PLS_Toolbox 9.0 (23592) Eigenvector Research, Inc., Manson, WA United States 98831). WiRE software was used for peak deconvolution, applying a baseline correction. The latter was used to deconvolve the Amide I subpeaks (1605, 1635, (1657), 1666, 1686 cm^−1^) and those present in the 2700–3800 cm^−1^ spectra (2943, 3220, 3333, 3457 cm^−1^). Models were built by measuring the average centers (intensity wavelength), width and Gaussian percentage of each subpeak derived from the deconvolution. The PLS toolbox automates the measurement of height and area of each peak of interest with a local baseline correction, after the manual creation of a model, according to the mean values of the peaks of interest from all spectra.

### 2.4 Protein extraction and enzyme-linked immunosorbent assay

Decorin (DCN) content was evaluated using the enzyme-linked immunosorbent assay (ELISA). For this purpose, the protein extraction was performed as previously described (Manon et al., 2022). Samples of each HFL and HP were weighted, cut and lysed with the buffer solution containing the radioimmunoprecipitation assay (RIPA, ThermoFisher Scientific, 89900), the protease inhibitor cocktail (Sigma-Aldrich, P8340-1 ML), and phosphatase inhibitors (PhosSTOP™, Roche, Basel, Switzerland). The samples were subjected to four cycles in the Precellys homogenizer (Bertin Technologies SAS, France) at 7,200 rpm. The protein concentration in the supernatant was determined with the Pierce™ BCA Protein assay kit (ThermoFisher Scientific, 23227) and normalized to the sample weight.

Subsequently, DCN content was evaluated using the RayBio^®^ Human Decorin ELISA kit (RayBiotech, ELH-Decorin-1,13485) following an adaptation of the manufacturer’s instructions. Briefly, since the protein concentration was low, larger volumes were used to achieve the required concentration per well. Therefore, 145 µL of tissue homogenates and standards were incubated in the pre-coated 96 well microplate at room temperature for 2.5 h. Following the washing steps, biotinylated antibody was added for 1 h. The subsequent washing step removed unbound antibodies before the addition of streptavidin conjugated horseradish peroxidase (HRP) solution. 5′-Tetramethylbenzidine (TMB) substrate was added after four washes and incubated protected from light. After 30 min, the reaction was stopped (Stop solution of 0.2 M sulfuric acid) and the plate was red at 450 nm. Standard curve was obtained using Sigmaplot 13 software (version 20, SPSS, Inc., Chicago, IL, United States) and results were expressed as pg of DCN per mg of total protein (pg/mg).

### 2.5 GAG quantification

The content of sulfated glycosaminoglycans (GAGs) was quantified in native and decellularized HP and HFL samples (*n* = 3) using the Blyscan Sulfated Glycosaminoglycan Assay (Biocolor LTD., Carrickfergus, Northern Ireland) and following the manufacturer’s protocol. Sulfated GAGs include chondroitin sulfate, dermatan sulfate, heparan sulfate and keratan sulfate. Three samples of 25 mg of each tissue were harvested. Samples were then first freeze-dried and dry-weighted. Before the quantitative measurement, each sample was placed in a Papain reagent (50 mL Na_2_HPO_4_/NaH_2_PO_4_ 0.2 M pH 6.4, 400 mg sodium acetate, 200 mg EDTA and 40 mg cysteine HCL, 250 µL papain) overnight at 65°C for GAG extraction. Following centrifugation (10 min, 10,000 rpm), 100 µL of each sample were taken and 1 mL of Dye Reagent was added for 30 min. The Eppendorfs were centrifuged again at 12,000 rpm for 10 min and the supernatant discarded. Dissociation reagent (500 µL) was added for 10 min and, after a final centrifugation (5 min, 12,000 rpm), 200 µL of each sample were transferred to a 96-well plate. Standards were also prepared according to the manufacturer’s protocol. The plate was read at 630 nm three times and the average of the three readings was used for final quantification. The concentration of GAGs was expressed in µg/mg dry weight.

### 2.6 Statistics

Statistical analyses were carried out using GraphPad Prism (Version 8.4.0, Inc., San Diego, CA, United States) and SPSS software (Version 27, IBM SPSS, Inc., Chicago, IL, United States). Data were expressed as the mean ± standard error of mean (SEM) and compared with the non-parametric Mann-Whitney or Kruskal-Wallis test because of the small dataset and the subsequent non-respect of normality (tested by Shapiro-Wilk test). Pairwise comparisons were performed using the Dunn’s test. All tests were two-tailed. The significance level was considered at a *p*-value *<* 0.05.

## 3 Results

All the decellularization protocols successfully achieved the Crapo’s criteria (Crapo et al., 2011). These standards include an absence of nuclei visible by histological analysis (DAPI, HE staining), a DNA quantity of less than 50 ng/mg dry weight and a length of less than 200 bp for persistent DNA fragments after treatment. In all the samples, DNA concentration felt below the critical threshold of 50 ng/mg of dry weight and no cellular traces were visible on microscopic analyses.

### 3.1 Weak alteration of collagen structure

**FIGURE 1:**
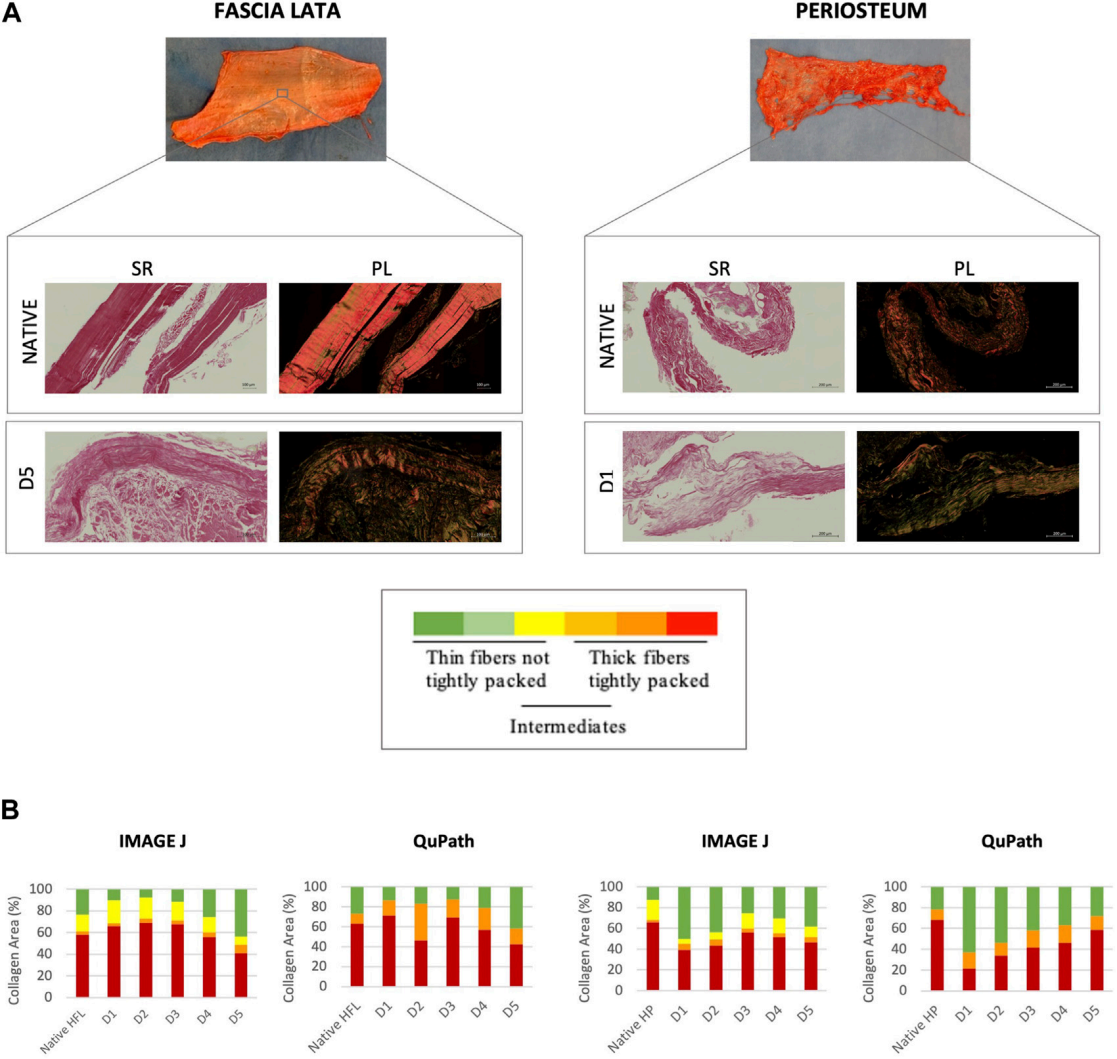
Qualitative **(A)** and quantitative **(B)** evaluations of the collagen organization of sections through native (*n* = 12) and decellularized HFL (*n* = 4) and HP (*n* = 3). **(A)** Sirius Red staining under bright field (SR, left) and polarized light (PL, right) obtained with ZEISS Axio Scan.Z1 Slide. Same analyses were carried out with polarized light microscope but are not shown. Variation in the polarization of collagen fibers under polarized light reflects differences in fiber thickness, density and packing: from green (thin, ≤0.8 µm, and loosely packed) to red (thick, 1.6–2.4 µm, and tightly packed) (Dayan et al., 1989). **(B)** Quantitative analyses were performed using ImageJ for the microscopic images and Qupath for scanner images. The quantitative analysis confirmed the qualitative assumptions: D5 and D1 protocols affected the most the HFL and HP samples respectively, with a higher average of thin fibers and lower of thick ones assessed by both ImageJ (microscope) and QuPath (scanner), despite remaining not significant.

HFL samples appeared to be more disorganized after protocol D5 treatment than the other subgroups, as highlighted by a decrease, although not significant, of thick (1.6–2.4 µm) red hue fibers (ImageJ: −29%; QuPath: −32.6%) and an increase in thin (≤0.8 µm) green ones (ImageJ: +87.9%; QuPath: +53.7%) (Figure 1). HP samples seemed to be more affected by protocol D1. Although not significant, the D1-treated tissues presented the lowest proportion of red fibers (ImageJ: −33.8%; QuPath: −68.3%) and the highest of green ones (ImageJ: +300%, QuPath: +189%) compared to natives. No significant differences in the amount of intermediate fibers (yellow/orange) were recorded.

### 3.2 Weak difference in the matrix molecular composition in native tissues

Raman spectroscopy revealed no statistical differences between the molecular composition of native HFL and HP. Both tissues presented similar amounts of hydroxyproline and collagen-bound water, although HFL samples showed a slightly higher proportion of enzymatic crosslinks (+13.6%, *p* > 0.05) and a slightly lower amount of non-enzymatic crosslinks (−55.8%, *p* > 0.05) than native HP.

### 3.3 Weak alteration of the matrix molecular composition in decellularized tissues

#### 3.3.1 Human fascia lata

Increase in tissue hydration was the most marked modification after HFL decellularization (Table 2), at the limit of significance for all three biomarkers of collagen-related water for all D-protocols: the hydroxyproline OH groups beside water bound to collagen (IR 3457/2943, *p* = 0.054), the NH groups beside organic matrix related water (IR 3333/2943, *p* = 0.052) and the organic matrix related water parameter (IR 3220/2943, *p* = 0.061). These increases were then confirmed, by a pairwise Dunn’s comparison, for the three biomarkers in protocols D3 (IR 3220/2943, *p* = 0.030; IR 3333/2943 *p* = 0.047 and IR 3457/2943, *p* = 0.037) and D5 (*p* = 0.030, *p* = 0.023 and *p* = 0.037 respectively) when compared to native tissues. An average loss of 25.2% (*p* = 0.195) in the amount of enzymatic crosslinks was also observed after the D5 treatment, which was the largest, though not significant, loss among all protocols. Hydroxyproline and pentosidine content were not significantly affected by the chemical treatments as well.

**TABLE 2:** Raman spectroscopy data of Human Fascia Lata matrices after decellularization with 5 different protocols (D1-D5) (*n* = 3/protocol and native). AGEs: Advanced Glycation End Products, NH: amine group, OH: hydroxide. Results are expressed as Mean ± SEM (Kruskal-Wallis).

		*Native*	*D1*	*D2*	*D3*	*D4*	*D5*	Kruskal-Wallis
								*p-value*
700–1800 cm^−1^	Hydroxyproline *875/920*	0.601 ± 0.031	0.600 ± 0.031	0.610 ± 0.045	0.530 ± 0.035	0.637 ± 0.092	0.589 ± 0.080	0.861
	Pentosidine (AGEs) *1342/920*	0.391 ± 0.068	0.365 ± 0.005	0.378 ± 0.039	0.384 ± 0.024	0.322 ± 0.033	0.338 ± 0.015	0.560
	Enzymatic Cross-links *1666/1686*	4.191 ± 0.430	3.435 ± 0.299	3.183 ± 0.142	3.210 ± 0.118	4.490 ± 0.737	3.136 ± 0.172	0.121
2700–3800 cm^−1^	Organic matrix related water 3220*/2943*	0.062 ± 0.016	0.285 ± 0.020	0.252 ± 0.085	0.446 ± 0.083	0.270 ± 0.052	0.377 ± 0.041	0.061
	NH groups beside organic matrix related water *3333/2943*	0.078 ± 0.005	0.141 ± 0.007	0.155 ± 0.027	0.194 ± 0.027	0.131 ± 0.028	0.196 ± 0.016	0.052
	OH groups of hydroxyproline next to collagen-related water *3457/2943*	0.005 ± 0.001	0.054 ± 0.004	0.039 ± 0.020	0.086 ± 0.017	0.055 ± 0.011	0.078 ± 0.008	0.054

#### 3.3.2 Human periosteum

HP samples hydration also increased with all decellularization protocols (Table 3) but in a less important way than HFL. D1-HP presented the lowest average of enzymatic crosslinks (−37.4%, *p* = 0.137) and pentosidine (−46.7%, *p* = 0.332) among the different decellularization protocols. Once again, the content of hydroxyproline did not significantly vary according to the treatment.

**TABLE 3:** Raman spectroscopy data of Human Periosteum matrices after decellularization with 5 different protocols (D1-D5) (n = 3/protocol and native). AGEs: Advanced Glycation End Products, NH: amine group, OH: hydroxide. Results are expressed as Mean ± SEM (Kruskal-Wallis).

		*Native*	*D1*	*D2*	*D3*	*D4*	*D5*	Kruskal-Wallis
								*p-value*
700–1800 cm^-1^	Hydroxyproline 875/920	0.922 ± 0.140	0.632 ± 0.043	0.599 ± 0.093	0.663 ± 0.038	0.626 ± 0.058	0.634 ± 0.042	0.544
	Pentosidine (AGEs) 1342/920	0.610 ± 0.111	0.325 ± 0.101	0.381 ± 0.055	0.417 ± 0 .115	0.399 ± 0.071	0.375 ± 0.016	0.460
	Enzymatic Cross-links 1666/1686	3.621 ± 0.099	2.269 ± 0.201	2.465 ± 0.220	2.328 ± 0.250	2.545 ± 0.061	2.346 ± 0.109	0.250
2700–3800 cm^-1^	Organic matrix related water 3220*/2943*	0.152 ± 0.094	0.414 ± 0.065	0.456 ± 0.217	0.927 ± 0.355	0.455 ± 0.179	0.463 ± 0.102	0.267
	NH groups beside organic matrix related water *3333/2943*	0.171 ± 0.050	0.234 ± 0.034	0.176 ± 0.058	0.370 ± 0.150	0.185 ± 0.059	0.223 ± 0.041	0.698
	OH groups of hydroxyproline next to collagen-related water *3457/2943*	0.006 ± 0.004	0.072 ± 0.020	0.084 ± 0.041	0.163 ± 0.054	0.087 ± 0.036	0.093 ± 0.021	0.100

#### 3.3.3 Irradiated human fascia lata

**FIGURE 2:**
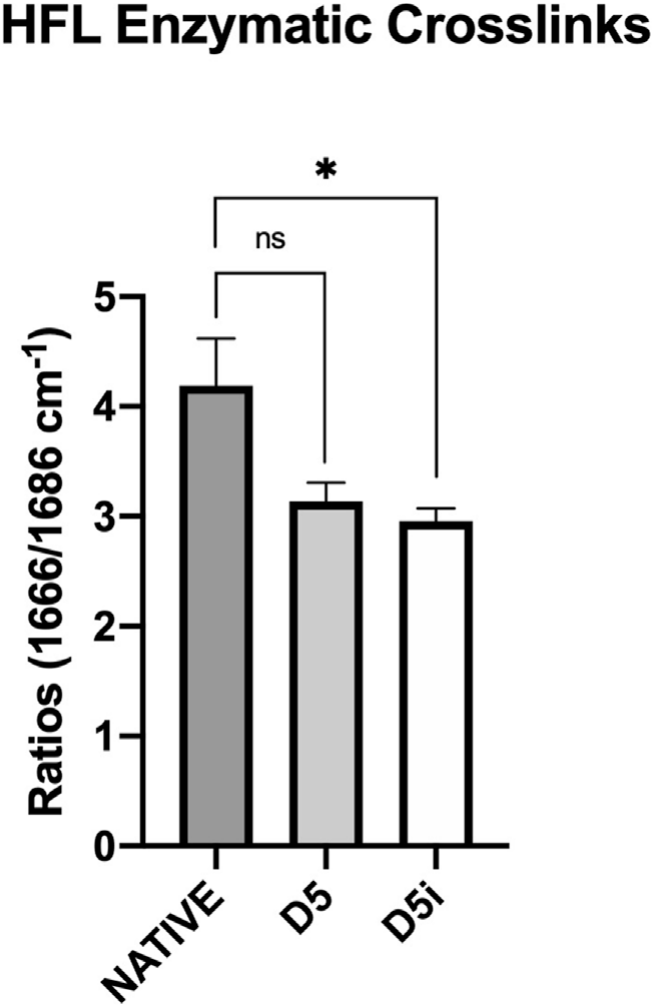
Measurement of enzymatic crosslinks by Raman spectroscopy in samples of native HFL and HFL decellularized according to the fifth protocol, followed (D5i) or not (D5) by gamma irradiation. The decellularization chemical treatment impaired the amount of enzymatic crosslinks but without significance (−25.2%, *p* = 0.195). However, after sterilization, the amount of enzymatic crosslink was further reduced and this reduction became significant (−29.4%, *p* = 0.037) compared to native.

By multiple comparison analyses, we highlighted a further loss in the proportion of enzymatic crosslinks in D5i sterilized samples, leading to a significant difference with natives (−29.4%, *p* = 0.037) (Figure 2). However, hydroxyproline and pentosidine levels were similar to that of native tissues (Table 4). Moreover, while hydration increased after decellularization (D1 to D5) of both tissues (HFL and HP), this proportion decreased after sterilization processes for HFL (D1i to D5i), without returning to basal levels of natives; the data obtained with protocol D5i remained the most different.

**TABLE 4:** Raman spectroscopy data of Human Fascia Lata matrix after decellularization with 5 different protocols followed by irradiation (D1i-D5i) (*n* = 3/protocol and native). AGEs: Advanced Glycation End Products, NH: amine group, OH: hydroxide. Results are expressed as Mean ± SEM (Kruskal-Wallis) and significant values are in bold-italic.

		*Native*	*D1i*	*D2i*	*D3i*	*D4i*	*D5i*	Kruskal-Wallis
								*p*-value
700–1800 cm^-1^	Hydroxyproline 875/920	0.601 ± 0.031	0.670 ± 0.086	0.586 ± 0.018	0.598 ± 0.036	0.607 ± 0.037	0.581 ± 0.077	0.942
	Pentosidine (AGEs) 1342/920	0.391 ± 0.068	0.384 ± 0.036	0.326 ± 0.015	0.320 ± 0.013	0.356 ± 0.024	0.345 ± 0.013	0.666
	Enzymatic Cross-links 1666/1686	4.191 ± 0.430	3.136 ± 0.162	3.134 ± 0.120	3.062 ± 0.090	3.273 ± 0.264	2.957 ± 0.116	0.161
2700–3800 cm^-1^	Organic matrix related water 3220*/2943*	0.062 ± 0.016	0.115 ± 0.006	0.102 ± 0.005	0.185 ± 0.065	0.119 ± 0.017	0.279 ± 0.037	0.058
	NH groups beside organic matrix related water *3333/2943*	0.078 ± 0.005	0.084 ± 0.004	0.086 ± 0.003	0.104 ± 0.022	0.086 ± 0.004	0.151 ± 0.023	0.145
	OH groups of hydroxyproline next to collagen-related water *3457/2943*	0.005 ± 0.001	0.020 ± 0.003	0.014 ± 0.0004	0.030 ± 0.013	0.020 ± 0.0006	0.048 ± 0.006	***0.034***

### 3.4 Interfibrillar bridges of decorin

**FIGURE 3:**
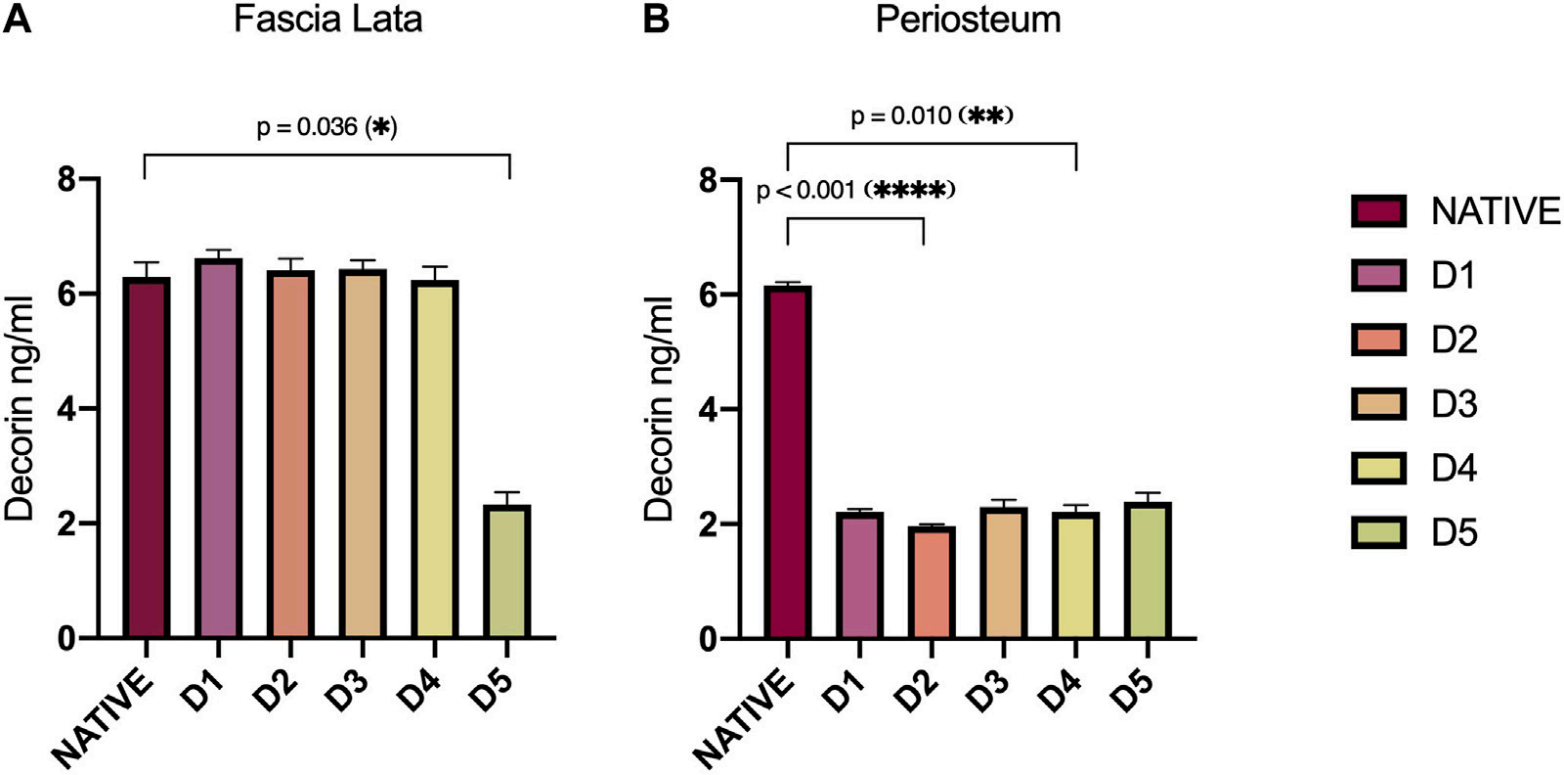
Decorin (DCN) levels (ng/mL) assessed by ELISA in HFL **(A)** and HP **(B)** protein extracts according to sample treatments (D1-D5). DCN in HFLs was significantly disrupted by tissue engineering processes in D5-treated tissue (*p* = 0.036). Concerning HP, DCN level was reduced with all decellularization protocols, but most importantly in D2 (*p* < 0.001) and D4 (*p* = 0.010)-treated tissues. Error bars represent the SEM. D: Decellularization protocol.

ELISA analyses of protein extracts from HP and HFL samples provide a deeper insight into the effect of chemical treatments on interfibrillar bridges (Figure 3). The DCN levels in HFL were approximately 6.40 ng/mL (±0.23) for all subgroups except D5-tissues, which averaged 2.3 ng/mL (±0.33) (*p* = 0.036). HP samples of all D-protocols presented a homogenous alteration of DCN levels compared with the native subgroup, with a mean of 2.2 ng/mL (±0.15) *versus* 6.2 ng/mL (±0.04) for natives. DCN levels in protocol D2 were the lowest, exhibiting a significant decrease compared to natives (*p* < 0.001), followed by protocol D4 (*p* = 0.010).

**FIGURE 4:**
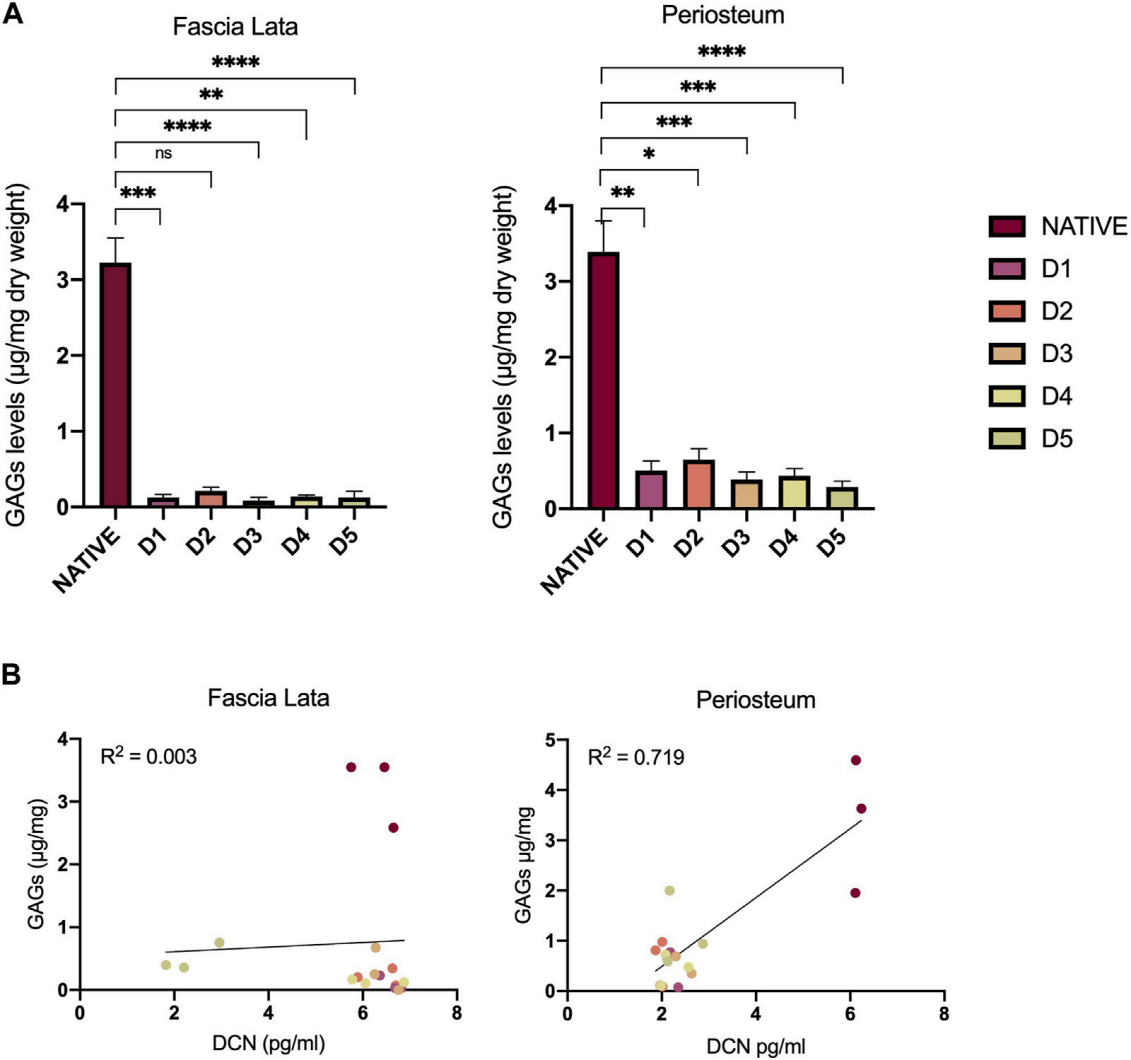
Impact of decellularization approaches on GAGs and DCN. **(A)** Quantitative analyses of GAG content in HFL and HP samples. The levels significantly decreased after all chemical treatments for both HFL (D1 (*p* = 0.0004), D2 (*p* = 0.024), D3 (*p* < 0.0001), D4 (*p* = 0.012), D5 (*p* < 0.0001)) and HP (D1 (*p* = 0.0002), D2 (*p* = 0.007), D3 (*p* < 0.0001), D4 (*p* < 0.0001), D5 (*p* = 0.007)) samples. **(B)** Variation of DCN and GAGs resulting from the chemical treatments are not related for both HFL (*R* ^2^ = 0.022) and HP (*R* ^2^ = 0.784). However, all decellurazed samples present similar profiles clearly distinguishable from native tissues. Error bars represent the standard error of the mean (SEM). GAGs: Glycosaminoglycans, D: Decellularization protocol, DCN: decorin.

Since interfibrillar bridges are formed by an association between DCN and sulfated GAG, we wanted to identify a relationship in their persistence within the tissue-engineered samples. In both tissue types, total sulfated GAG levels were similarly and significantly decreased after all D-protocols (Figure 4A). HFL decellularized samples contained average levels of 0.24 µg of GAG per mg of dry weight (±0.20), while native sample levels averaged 3.32 μg/mg (±0.38). For HP samples, a similar ratio was found, with an average of 0.66 μg/mg (±0.19) for decellularized tissues and 3.06 μg/mg (±0.79) for native tissues. No correlation was observed between GAG and DCN levels for both HFL (*R* ^2^ = 0.022) and HP (*R* ^2^ = 0.784) samples (Figure 4B). Changes in DCN and GAG level, resulting from chemical treatments were not related.

## 4 Discussion

None of the five decellularization protocols (D1 to D5, Table 1) significantly altered collagen arrangement. Detergent-free treatment (D5) disorganized HFL samples to a greater extent, significantly reducing DCN and GAGs, and non-significantly reducing collagen cross-links. HP samples, treated with detergent-based treatment D1, showed collagen disorganization, and a reduction in enzymatic cross-links. Overall, decellularization had minimal impact on the matrix, while the addition of gamma irradiation impact leads to a significant negative effect on the integrity of collagen cross-links.

Structural modifications were therefore most pronounced in HFL when treated with the D5 protocol and in HP with the D1 protocol. This was first observed with the assessment of collagen bundle birefringence using PLM to understand the impact of the engineered treatments on the architectural arrangement (Dayan et al., 1989; Ribeiro et al., 2013; Rittié, 2017). The robustness of the results is confirmed by the convergence of birefringence measurements between various analysis tools. Interestingly, each of these subgroups, HFL-D5 and HP-D1, also showed the lowest levels of enzymatic crosslinks as measured by Raman spectroscopy. In the literature, optical density measures under polarized light were already used as indicator of crosslinked collagen organization (Kirby, Heuerman, and Yellon, 2018; Lien, Chen, and Phan, 2022).

Previous studies confirmed collagen persistence in decellularized scaffolds (Duisit et al., 2017; Manon et al., 2023). Being the major ECM component, collagen biological and architectural functions have already been well characterized (Snedeker and Gautieri, 2014; Dong and Lv, 2016). Ensuring the persistence of enzymatic crosslinks in decellularized collagen scaffolds is therefore crucial for maintaining the functional properties of native matrices, contributing to fibril stability and improving tissue biomechanical competence. Therefore, physical and chemical methods are nowadays used to achieve intermolecular collagen crosslinking in engineered scaffolds (Dong and Lv, 2016).

In addition to collagen enzymatic crosslinks, its overall molecular composition has an impact on its structure and biomechanical properties. Indeed, AGEs are known to alter the mechanical strength of tissues (Saito and Marumo, 2010). In addition, as Unal and Akkus (2015) have shown, the hydration level of bones also plays a role in their mechanical properties. Moreover, it is also known that physical training has an impact on collagen crimps, which can be analyzed by measuring birefringence (Mazon et al., 2018). Therefore, by taking all collagen parameters into account, we could in future investigate a relationship between the structural data of our scaffolds and their mechanical properties.

It is important to note, however, that the samples were taken from old subjects, which may also have an impact on the results. Since the rate of crosslinks is age-related, the slight decrease in enzymatic crosslinks after the engineering protocols could be explained by an age-related decrease, itself associated with a decrease in the mechanical capacity of the tissue. These decreases could therefore possibly be significant in tissues from younger subjects, which should be verified in the future. Moreover, it should be noted that these analyses were carried out on a small number of samples randomly taken from the entire decellularized scaffold, allowing potential tissue heterogeneity to be taken into account.

Raman spectra of the HFL samples differed slightly from those of the periosteal ones, particularly in the amide I region (1670/1690 cm^−1^). We suspected interference from tissues other than periosteum, adding some background in the spectra. The samples were harvested from old subjects and, despite efforts to remove muscle and fat, some residues remained due to the thinness of the HP. Indeed, anomalies in the spectra were observed for all protocols, including native tissue, with the exception of protocol D5, the only one to include a degreasing step. Therefore, in line with the literature, an additional peak (1657 cm^−1^) was added for deconvolution of this amide region, known to correct for potential interference from lipid vibrations (Mandair et al., 2020). In addition, we observed increased hydration in samples after decellularization, probably because decellularization disrupts the natural barriers regulating hydration. This leads to increased water uptake during abundant rinsing steps. Irradiation, however, leads to a partial loss of this increased hydration, without returning to baseline levels.

Since these decellularized tissues are intended to regenerate *in vivo* damaged structures, sterilization processes are required to prevent pathogen transmission (Dziedzic-Goclawska et al., 2005; Seto, Gatt, and Dunn, 2008; Harrell et al., 2018). Althoug gamma irradiation is commonly used in bone banks for sterilization, it causes radiolysis of water molecules, releasing free radicals (Nguyen et al., 2007) that damage collagen (Seto, Gatt, and Dunn, 2008; Harrell et al., 2018). Previous studies on bones showed a negative synergic effect on mechanical resistance when irradiation was added to freeze-dried samples (Cornu et al., 2000; Cornu et al., 2011), attributed to a significant reduction in enzymatic crosslinks density (Cornu, 2012). This is consistent with our results revealing a more pronounced alteration of crosslinks post-irradiation. Future studies should explore decellularization procedures under totally sterile conditions to avoid the detrimental effects of subsequent irradiation on tissue properties.

We did not investigate irradiated periosteum since the priority, for the creation of the new regenerative membrane, was the use of irradiated decellularized HFL (Manon et al., 2023).

DCN-GAG complexes are essential for the ECM organization, especially in collagen fibrillogenesis, as evidenced by the alteration of the latter in DCN-deficient mice (Gubbiotti et al., 2016; Zhang et al., 2018). This PG is also involved in stress transfer between collagen fibrils (Thorpe, 2010) and in the bioavailability of growth factors such as TGF-β (Gubbiotti et al., 2016; Zhang et al., 2018). Alteration in decorin content vary according to the tissue type, with HFL samples globally maintaining their DCN concentration after decellularization, except for D5, while HP samples show a decrease in all D-protocols.

Furthermore, it has already been demonstrated that detergent-based decellularization affects the GAG composition of ECM (Duisit et al., 2017; Liguori et al., 2020; Uhl et al., 2020), as confirmed by our quantitative measurements, with potential dysfunction of persistent GAGs (Uhl et al., 2020). However, the maintenance of functional GAGs is necessary. Abundantly distributed on cell surface and in the ECM, they modulate various physiological processes. They provide biochemical and mechanical support in the ECM and influence cell behavior (adhesion, proliferation, differentiation) (Salbach et al., 2012; Menezes et al., 2022).

Despite the lower proportions of DCN and GAG in decellularized samples compared to native ones, no correlation could be demonstrated in their alteration after chemical treatments. Since GAGs are ubiquitous on the cell surface, their removal by the decellularization protocol is expected. Therefore, proportionally, the loss of GAGs could be greater than that of DCN.

The present study confronts different decellularization approaches. Protocols D1 to D4 are based on the use of Triton, SDS and DNase and ensure cell membrane lysis, protein solubilization and residual DNA elimination. Triton and SDS are known for their efficiency in decellularization, but also for altering the structure of the ECM (Crapo et al., 2011). Protocol D5 consists of a succession of solvent- and detergent-based baths, which has also already demonstrated its decellularization potential (Manon et al., 2023), including the monitoring of Crapo’s criteria (Crapo et al., 2011). In this protocol, solvents such as ethanol and acetone are used for cell lysis and lipid removal but are both also known as altering the ECM ultrastructure. The additional advantage of this protocol is that it also neutralizes prions, which the other protocols do not allow.

As qualitatively shown in the Figure 1 on the SR stained sections, more undulating collagen structure are observed in decellularized tissues than in native tissues. The crosslink alteration could modifiy the fibers alignment. In the future, analysis of the collagen distribution could support the observations made in this study. Approaches such as transmission electron microscopy (Adeoye et al., 2022), histology and contrast-enhanced microCT (Maes et al., 2022) could be used and be gathered in order to precisely cartograph the entire HFL structure.

In conclusion, a relationship was observed between the ECM structure and its molecular composition. The decellularization approaches demonstrated no significant alteration of the matrices, which justifies, along with the analysis of other parameters, the future clinical use of these processed matrices for bone regeneration. However, the combination of D5 treatment with subsequent irradiation appears the most deleterious for tissue properties. In the future, an analysis of the entire sample surface would provide more accurate information about potential sample heterogeneity, and precise quantitative analyses of the crosslink content could further validate our results. It would also be interesting to correlate all the observations of this study with the mechanical properties of the tissues.

## Data Availability

The original contributions presented in the study are included in the article/Supplementary material, further inquiries can be directed to the corresponding authors.

## References

[B1] Adeoye A. O. Mukasheva F. Smatov S. Khumyrzakh B. Kadyr S. Shulgau Z. (2022). A biomimetic synthetic nanofiber-based model for anterior cruciate ligament regeneration. Front. Bioeng. Biotechnol. 10, 969282. 10.3389/fbioe.2022.969282 36394020 PMC9644221

[B2] Aktuglu K. Erol K. Vahabi A. (2019). 'Ilizarov bone transport and treatment of critical-sized tibial bone defects: a narrative review. J. Orthop. Traumatol. 20, 22. 10.1186/s10195-019-0527-1 30993461 PMC6468024

[B3] Blottner D. Huang Y. Trautmann G. Sun L. (2019). The fascia: continuum linking bone and myofascial bag for global and local body movement control on Earth and in Space. A scoping review. REACH 14-15, 100030. 10.1016/j.reach.2019.100030

[B4] Chen K. Lin X. Zhang Q. Ni J. Li J. Xiao J. (2015). 'Decellularized periosteum as a potential biologic scaffold for bone tissue engineering. Acta Biomater. 19, 46–55. 10.1016/j.actbio.2015.02.020 25725472

[B5] Cornu O. (2012). “Influence of freeze-drying and irradiation on mechanical properties of human cancellous bone: application to impaction bone grafting,” in Bone grafting. Editor Alessandro Zorzi Dr (Croatia: InTech), 41–58. Chapter 3.

[B6] Cornu O. Banse X. Docquier P. L. Luyckx S. Delloye C. (2000). 'Effect of freeze-drying and gamma irradiation on the mechanical properties of human cancellous bone. J. Orthop. Res. 18, 426–431. 10.1002/jor.1100180314 10937629

[B7] Cornu O. Boquet J. Nonclercq O. Docquier P. L. Van Tomme J. Delloye C. (2011). 'Synergetic effect of freeze-drying and gamma irradiation on the mechanical properties of human cancellous bone. Cell Tissue Bank. 12, 281–288. 10.1007/s10561-010-9209-1 20703816

[B8] Costa-Almeida R. Gonçalves A. Gershovich P. Rodrigues M. Reis R. L. Gomes M. (2015). Tendon stem cell niche.

[B9] Crapo P. M. Gilbert T. W. Badylak S. F. (2011). An overview of tissue and whole organ decellularization processes. Biomaterials 32, 3233–3243. 10.1016/j.biomaterials.2011.01.057 21296410 PMC3084613

[B10] Dalisson B. Charbonnier B. Ahmed A. Gilardino M. Harvey E. Makhoul N. (2021). 'Skeletal regeneration for segmental bone loss: vascularised grafts, analogues and surrogates. Acta Biomater. 136, 37–55. 10.1016/j.actbio.2021.09.053 34626818

[B11] Dayan D. Hiss Y. Hirshberg A. Bubis J. J. Wolman M. (1989). Are the polarization colors of picrosirius red-stained collagen determined only by the diameter of the fibers? Histochemistry 93, 27–29. 10.1007/bf00266843 2482274

[B12] Delloye C. van Cauter M. Dufrane D. Francq B. G. Docquier P. L. Cornu O. (2014). Local complications of massive bone allografts: an appraisal of their prevalence in 128 patients. Acta Orthop. Belg 80 (2), 196–204. 25090792

[B13] Dong C. Lv Y. (2016). Application of collagen scaffold in tissue engineering: recent advances and new perspectives. Polym. (Basel) 8, 42. 10.3390/polym8020042 PMC6432532 30979136

[B14] Duchamp de Lageneste O. Colnot C. (2019). Periostin in bone regeneration. Adv. Exp. Med. Biol. 1132, 49–61. 10.1007/978-981-13-6657-4_6 31037624

[B15] Duchamp de Lageneste O. Julien A. Abou-Khalil R. Frangi G. Carvalho C. Cagnard N. (2018). 'Periosteum contains skeletal stem cells with high bone regenerative potential controlled by Periostin. Nat. Commun. 9, 773. 10.1038/s41467-018-03124-z 29472541 PMC5823889

[B16] Duisit J. Maistriaux L. Taddeo A. Orlando G. Joris V. Coche E. (2017). 'Bioengineering a human face graft: the matrix of identity. Ann. Surg. 266, 754–764. 10.1097/sla.0000000000002396 28742686

[B17] Dwek J. R. (2010). The periosteum: what is it, where is it, and what mimics it in its absence? Skelet. Radiol. 39, 319–323. 10.1007/s00256-009-0849-9 PMC2826636 20049593

[B18] Dziedzic-Goclawska A. Kaminski A. Uhrynowska-Tyszkiewicz I. Stachowicz W. (2005). 'Irradiation as a safety procedure in tissue banking. Cell Tissue Bank. 6, 201–219. 10.1007/s10561-005-0338-x 16151960

[B19] Evans S. F. Chang H. Knothe Tate M. L. (2013). Elucidating multiscale periosteal mechanobiology: a key to unlocking the smart properties and regenerative capacity of the periosteum? Tissue Eng. Part B Rev. 19, 147–159. 10.1089/ten.teb.2012.0216 23189933 PMC3589889

[B20] Gautieri A. Redaelli A. Buehler M. J. Vesentini S. (2014). 'Age- and diabetes-related nonenzymatic crosslinks in collagen fibrils: candidate amino acids involved in Advanced Glycation End-products. Matrix Biol. 34, 89–95. 10.1016/j.matbio.2013.09.004 24060753

[B21] Gjaltema R. A. Bank R. A. (2017). 'Molecular insights into prolyl and lysyl hydroxylation of fibrillar collagens in health and disease. Crit. Rev. Biochem. Mol. Biol. 52, 74–95. 10.1080/10409238.2016.1269716 28006962

[B22] Gubbiotti M. A. Vallet S. D. Ricard-Blum S. Iozzo R. V. (2016). 'Decorin interacting network: a comprehensive analysis of decorin-binding partners and their versatile functions. Matrix Biol. 55, 7–21. 10.1016/j.matbio.2016.09.009 27693454 PMC6938589

[B23] Halper J. (2014). “Proteoglycans and diseases of soft tissues,” in Progress in heritable soft connective tissue diseases. Editor Halper J. (Dordrecht: Springer Netherlands).

[B24] Harrell C. R. Djonov V. Fellabaum C. Volarevic V. (2018). 'Risks of using sterilization by gamma radiation: the other side of the coin. Int. J. Med. Sci. 15, 274–279. 10.7150/ijms.22644 29483819 PMC5820857

[B25] Hussey G. S. Dziki J. L. Badylak S. F. (2018). Extracellular matrix-based materials for regenerative medicine. Nat. Rev. Mater. 3, 159–173. 10.1038/s41578-018-0023-x

[B26] Julien A. Perrin S. Martínez-Sarrà E. Kanagalingam A. Carvalho C. Luka M. (2022). 'Skeletal stem/progenitor cells in periosteum and skeletal muscle share a common molecular response to bone injury. J. bone mineral Res. official J. Am. Soc. Bone Mineral Res. 37, 1545–1561. 10.1002/jbmr.4616 PMC9543664 35652423

[B27] Kamml J. Ke C. Y. Acevedo C. Kammer D. S. (2023). The influence of AGEs and enzymatic cross-links on the mechanical properties of collagen fibrils. J. Mech. Behav. Biomed. Mater 43, 105870. 10.1016/j.jmbbm.2023.105870 PMC11522032 37156073

[B28] Kirby M. A. Heuerman A. C. Yellon S. M. (2018). 'Utility of optical density of picrosirius red birefringence for analysis of cross-linked collagen in remodeling of the peripartum cervix for parturition. Integr. Gynecol. Obstet. J. 1. 10.31038/IGOJ.2018107 PMC6117116 30175325

[B29] Lien C. H. Chen Z. H. Phan Q. H. (2022). 'Birefringence effect studies of collagen formed by nonenzymatic glycation using dual-retarder Mueller polarimetry. J. Biomed. Opt. 27, 087001. 10.1117/1.jbo.27.8.087001 36452033 PMC9349470

[B30] Liguori G. R. Liguori T. T. A. de Moraes S. R. Sinkunas V. Terlizzi V. van Dongen J. A. (2020). 'Molecular and biomechanical clues from cardiac tissue decellularized extracellular matrix drive stromal cell plasticity. Front. Bioeng. Biotechnol. 8, 520. 10.3389/fbioe.2020.00520 32548106 PMC7273975

[B31] Lin X. Zhao C. Zhu P. Chen J. Yu H. Cai Y. (2018). 'Periosteum extracellular-matrix-mediated acellular mineralization during bone formation. Adv. Healthc. Mater 7. 10.1002/adhm.201700660 29266835

[B32] Lou Y. Wang H. Ye G. Li Y. Liu C. Yu M. (2021). 'Periosteal tissue engineering: current developments and perspectives. Adv. Healthc. Mater 10, e2100215. 10.1002/adhm.202100215 33938636

[B33] Maes A. Pestiaux C. Marino A. Balcaen T. Leyssens L. Vangrunderbeeck S. (2022). Cryogenic contrast-enhanced microCT enables nondestructive 3D quantitative histopathology of soft biological tissues. Nat. Commun. 13 (1), 6207. 10.1038/s41467-022-34048-4 36266273 PMC9584947

[B34] Mandair G. S. Oest M. E. Mann K. A. Morris M. D. Damron T. A. Kohn D. H. (2020). Radiation-induced changes to bone composition extend beyond periosteal bone. Bone Rep. 12, 100262. 10.1016/j.bonr.2020.100262 32258252 PMC7125315

[B35] Manon J. Evrard R. Fievé L. Bouzin C. Magnin D. Xhema D. (2023). 'A new osteogenic membrane to enhance bone healing: at the crossroads between the periosteum, the induced membrane, and the diamond concept. Bioengineering 10, 143. 10.3390/bioengineering10020143 36829637 PMC9952848

[B36] Manon J. Evrard R. Maistriaux L. Fievé L. Heller U. Magnin D. (2022). Periosteum and fascia lata: are they so different? Front. Bioeng. Biotechnol. 10, 944828. 10.3389/fbioe.2022.944828 36338112 PMC9627508

[B37] Masquelet A. C. Begue T. (2010). 'The concept of induced membrane for reconstruction of long bone defects. Orthop. Clin. North Am. 41, 27–37. 10.1016/j.ocl.2009.07.011 19931050

[B38] Mazon J. de Aro A. A. Simões P. W. Pimentel E. R. (2018). 'Effect of different resistance-training protocols on the extracellular matrix of the calcaneal tendon of rats. Ann. Anat. 216, 75–81. 10.1016/j.aanat.2017.11.002 29229272

[B39] Menezes R. Vincent R. Osorno L. Hu P. Treena Livingston Arinzeh (2022). Biomaterials and tissue engineering approaches using glycosaminoglycans for tissue repair: lessons learned from the native extracellular matrix. Acta Biomater. 163, 210–227. 10.1016/j.actbio.2022.09.064 36182056 PMC10043054

[B65] Nguyen H. Morgan D. A. Forwood M. R. (2007). Sterilization of allograft bone: effects of gamma irradiation on allograft biology and biomechanics. Cell Tissue Bank 8 (2), 93–105. 10.1007/s10561-006-9020-1 17063262

[B40] Pancheri F. Q. Eng C. M. Lieberman D. E. Biewener A. A. Dorfmann L. (2014). 'A constitutive description of the anisotropic response of the fascia lata. J. Mech. Behav. Biomed. Mater. 30, 306–323. 10.1016/j.jmbbm.2013.12.002 24361935

[B41] Paul R. G. Bailey A. J. (1996). 'Glycation of collagen: the basis of its central role in the late complications of ageing and diabetes. Int. J. Biochem. Cell Biol. 28, 1297–1310. 10.1016/s1357-2725(96)00079-9 9022289

[B42] Perrin S. Colnot C. (2022). 'Periosteal skeletal stem and progenitor cells in bone regeneration. Curr. Osteoporos. Rep. 20, 334–343. 10.1007/s11914-022-00737-8 35829950

[B43] Pritchard J. M. Willett T. L. (2017). “Pentosidine as a biomarker for poor bone quality and elevated fracture risk,” in Biomarkers in bone disease. Editors Patel V. B. Preedy V. R. (Dordrecht: Springer Netherlands).

[B44] Rappu P. M Salo A. Myllyharju J. Heino J. (2019). Role of prolyl hydroxylation in the molecular interactions of collagens. Essays Biochem. 63, 325–335. 10.1042/ebc20180053 31350381 PMC6744578

[B45] Ribeiro J. F. dos Anjos E. H. Mello M. L. de Campos Vidal B. (2013). 'Skin collagen fiber molecular order: a pattern of distributional fiber orientation as assessed by optical anisotropy and image analysis. PLoS One 8, e54724. 10.1371/journal.pone.0054724 23349957 PMC3548803

[B46] Rittié L. (2017). 'Method for picrosirius red-polarization detection of collagen fibers in tissue sections. Methods Mol. Biol. 1627, 395–407. 10.1007/978-1-4939-7113-8_26 28836216

[B47] Sainio A. Järveläinen H. (2020). Extracellular matrix-cell interactions: focus on therapeutic applications. Cell. Signal. 66, 109487. 10.1016/j.cellsig.2019.109487 31778739

[B48] Saito M. Marumo K. (2010). 'Collagen cross-links as a determinant of bone quality: a possible explanation for bone fragility in aging, osteoporosis, and diabetes mellitus. Osteoporos. Int. 21, 195–214. 10.1007/s00198-009-1066-z 19760059

[B49] Saito M. Marumo K. (2015). 'Effects of collagen crosslinking on bone material properties in health and disease. Calcif. Tissue Int. 97, 242–261. 10.1007/s00223-015-9985-5 25791570

[B50] Salbach J. TilmanRachner D. M. R. Ute Hempel U. A. Sandra Franz J.-C. S. Lorenz C. H. Franz S. (2012). Regenerative potential of glycosaminoglycans for skin and bone. J. Mol. Med. 90, 625–635. 10.1007/s00109-011-0843-2 22187113

[B51] Scott J. E. (2003). Elasticity in extracellular matrix shape modules of tendon, cartilage etc A sliding proteoglycan-filament model. J. physiology 553, 335–343. 10.1113/jphysiol.2003.050179 PMC2343561 12923209

[B52] Seto A. Gatt C. J. Jr. Dunn M. G. (2008). 'Radioprotection of tendon tissue via crosslinking and free radical scavenging. Clin. Orthop. Relat. Res. 466, 1788–1795. 10.1007/s11999-008-0301-9 18512113 PMC2584246

[B53] Shoulders M. D. Raines R. T. (2009). 'Collagen structure and stability. Annu. Rev. Biochem. 78, 929–958. 10.1146/annurev.biochem.77.032207.120833 19344236 PMC2846778

[B54] Snedeker J. G. Gautieri. A. (2014). The role of collagen crosslinks in ageing and diabetes - the good, the bad, and the ugly. Muscles, ligaments tendons J. 4, 303–308. 10.32098/mltj.03.2014.07 25489547 PMC4241420

[B55] Squier C. A. Ghoneim S. Kremenak C. R. (1990). 'Ultrastructure of the periosteum from membrane bone. J. Anat. 171, 233–239. 2081707 PMC1257144

[B56] Theocharis A. D. Skandalis S. S. Gialeli C. Karamanos N. K. (2016). Extracellular matrix structure. Adv. Drug Deliv. Rev. 97, 4–27. 10.1016/j.addr.2015.11.001 26562801

[B57] Thorpe C. T. (2010). Extracellular matrix synthesis and degradation in functionally distinct tendons. University College London.

[B58] Uhl F. E. Zhang F. Pouliot R. A. Uriarte J. J. Rolandsson Enes S. Han X. (2020). 'Functional role of glycosaminoglycans in decellularized lung extracellular matrix. Acta Biomater. 102, 231–246. 10.1016/j.actbio.2019.11.029 31751810 PMC8713186

[B59] Unal M. Akkus O. (2015). 'Raman spectral classification of mineral- and collagen-bound water's associations to elastic and post-yield mechanical properties of cortical bone. Bone 81, 315–326. 10.1016/j.bone.2015.07.024 26211992 PMC4640992

[B60] van Steenberghe M. Schubert T. Guiot Y. Bouzin C. Bollen X. Gianello P. (2017). 'Enhanced vascular biocompatibility of decellularized xeno-/allogeneic matrices in a rodent model. Cell Tissue Bank. 18, 249–262. 10.1007/s10561-017-9610-0 28238108

[B61] Vidal L. Kampleitner C. Brennan M. Á. Hoornaert A. Layrolle P. (2020). Reconstruction of large skeletal defects: current clinical therapeutic strategies and future directions using 3D printing. Front. Bioeng. Biotechnol. 8, 61. 10.3389/fbioe.2020.00061 32117940 PMC7029716

[B62] Yamauchi M. Sricholpech M. (2012). 'Lysine post-translational modifications of collagen. Essays Biochem. 52, 113–133. 10.1042/bse0520113 22708567 PMC3499978

[B63] Zhang W. Ge Y. Cheng Q. Zhang Q. Fang L. Zheng J. (2018). Decorin is a pivotal effector in the extracellular matrix and tumour microenvironment. Oncotarget 9, 5480–5491. 10.18632/oncotarget.23869 29435195 PMC5797066

[B64] Zhang X. Xie C. Lin A. S. Ito H. Awad H. Lieberman J. R. (2005). 'Periosteal progenitor cell fate in segmental cortical bone graft transplantations: implications for functional tissue engineering. J. bone mineral Res. official J. Am. Soc. Bone Mineral Res. 20, 2124–2137. 10.1359/jbmr.050806 PMC4527562 16294266

